# Isolation of a novel bio-peptide from walnut residual protein inducing apoptosis and autophagy on cancer cells

**DOI:** 10.1186/s12906-015-0940-9

**Published:** 2015-11-23

**Authors:** Sihui Ma, Di Huang, Mengxin Zhai, Lubing Yang, Sen Peng, Changxu Chen, Xiaoru Feng, Qiang Weng, Bolin Zhang, Meiyu Xu

**Affiliations:** College of Biological Science and Technology, Beijing Forestry University, Beijing, 100083 People’s Republic of China; Beijing Key Laboratory of Forest Food Processing and Safety, Beijing Forestry University, Beijing, 100083 China

**Keywords:** Walnut residual protein, Bio-peptide, Apoptosis, Autophagy, Cancer cells

## Abstract

**Background:**

Walnut is unique because they have a perfect balance of n-6 and n-3 polyunsaturated fatty acids. The increasing market demand of walnut lipids results in the large amount of the oil extraction residue. The walnut residue is rich in nutritional proteins, and the uneconomic use of the by-product discouraged the development of walnut industry. Anticancer peptides have recently received attention as alternative chemotherapeutic agents that overcome the limits of current drugs. The aim of this study was to investigate whether anticancer bioactive peptide is contained in walnut.

**Methods:**

Walnut residual protein was hydrolyzed separately by five different proteases. The sequential purification of the hydrolysates was carried out by ultra-filtration, gel filtration chromatography and RP-HPLC to obtain a cancer cell growth inhibitory peptide. Cell cycle distribution, Annexin V-FITC/PI double staining, TUNEL assay, western blot and immunofluorescence for LC3-II assay were used to detect apoptosis and autophagy on cells. Cytokine production was measured by ELISA kits, macrophage phagocytosis was measured by neutral red uptake assay, nitric oxide production was measured by Griess reagent.

**Results:**

The hydrolysates of walnut residual protein produced by papain under the optimal conditions (5 % substrate concentration and an enzyme-substrate ratio of 10 % at temperature 60 C for 3 h), showed significant growth inhibitory activity on MCF-7. The amino acid sequence of the purified peptide was identified as CTLEW with a molecular weight of 651.2795 Da. It is a novel bio-peptide with an amphiphilic structure. CTLEW induced both apoptosis and autophagy on MCF-7 cells, inhibited the cancer cells growth of Caco-2 and HeLa significantly, but did not show any cytotoxic activity against non-cancerous IEC-6 cells. Moreover, the bio-peptide enhanced proliferation and IL-2 secretion of spleen lymphocytes, promoted phagocytosis and NO production of macrophages.

**Conclusion:**

These results suggested that a novel bio-peptide, CTLEW inducing apoptosis and autophagy on MCF-7 cells can be released from walnut residual protein through papain hydrolyzing under the certain condition. The bio-peptide shows selective inhibition towards cancer cells growth and immunomodulatory activity.

## Background

Walnut (*Juglans regia* L.) is the most widespread tree nut in the world, which has been reported with keratolytic, antifungal, hypoglycemic, hypotensive, anti-oxidant, and sedative activities [1, 2]. Walnut is nutrient-dense food, mainly owing to its fat content as well as protein, vitamin and mineral profiles. Walnut is unique because they have a perfect balance of n-6 and n-3 polyunsaturated fatty acids, a ratio of 4:1, which has been shown to decrease the incidence of cardiovascular risk. Thus, walnut oil is extracted in large quantities. The increasing market demand of walnut lipids results in the large amount of the oil extraction residue. The walnut residue is rich in nutritional proteins, 450 g/kg on average [3]. However, it is used as forage usually, and the uneconomic use of the by-product discouraged the development of walnut industry. It is essential to improve the economic value of the walnut by-products [4], while elucidating the biological activities of the walnut protein are generally thought to be beneficial for effectively utilizing the by-product of walnut. Walnut protein has been reported to have antioxidant effect and can inhibit the activity of angiotensin I-converting enzyme (ACE), a dipeptidyl carboxypeptidase associated with the regulation of blood pressure as well as cardiovascular function [2, 5]. However, whether it has anticancer function is unknown.

Cancer is a major cause of mortality worldwide and cancer incidents has been rapidly increasing in recent years. The use of conventional chemotherapeutic agents that typically target rapidly dividing cancer cells is often associated with deleterious side effects due to drug-induced damage to normal cells and tissues [6]. Moreover, cancer cells develop resistance to these drugs that is mediated by the over expression of multidrugresistance proteins that pump the drugs out of cells and thus render the drugs ineffective [7]. Therefore, the research and development of more effective and less toxic anticancer agents has become necessary. Anticancer peptides have recently received attention as alternative chemotherapeutic agents that overcome the limits of current drugs. A growing body of evidence has shown that peptides from milk and soy proteins can prevent cancer [8, 9]. Anticancer effects also have been demonstrated in enzymatic protein hydrolysates (or peptides) of rapeseed, solitary tunicate and rice bran [10–12]. A 440.9 Da anchovy hydrophobic peptide was found to be able to induce apoptosis in human U937 lymphoma cells by increasing caspase-3 and caspase-8 activity [13]. Epinecidin-1, a peptide from fish (Epinephelus coioides) showed an antitumor effect similar to lytic peptides in human fibrosarcoma cells [14]. Whether anticancer bioactive peptide is contained in the walnut was not reported yet.

Peptides generated by the digestion of various proteins, from both animal and plant sources, possess biofunctional activity. These peptides are inactive within the sequences of their parent proteins and are released by proteolytic hydrolysis using commercially available enzymes or proteolytic microorganisms and fermentation methods [15, 16]. For instance, antioxidant peptides isolated from rapeseed proteins 3 and angiotensin-I-converting enzyme (ACE) inhibitory peptides Gly-Pro-Leu and Gly-Pro-Met extracted from the skin of Theragra chalcogramma 4 are natural peptides [17]. Once such bioactive peptides are liberated depending on their structural, compositional, and sequential properties, they may exhibit various biofunctional activities,because bioactive peptides can be absorbed in the intestine and enter the blood stream directly, which ensures their bioavailability in vivo and a physiological effect at the target site [17]. Different enzymes give the hydrolysates different qualities and bioactivities, since enzymes have specific cleavage positions on polypeptide chains, protein hydrolysates prepared with different enzymes exhibit different amino acid sequences and peptide lengths [18]. For example, walnut protein showed potent antioxidant activity after being hydrolyzed by pepsin [2], while it will reveal significant ACE inhibitory activity by neutral proteinase [5]. Various parameters also play a great role in hydrolyzing protein, such as Temperature, time, enzyme-substrate ratio and substrate concentration. Study on how to prepare the walnut protein hydrolysate with anticancer activity has not been reported so far.

In the present study, walnut residual protein was hydrolyzed separately by five different proteases to find a suitable enzyme which can release anticancer peptides, and then the hydrolysis condition of this enzyme on the walnut residual protein was optimized. Anticancer peptide was isolated from the walnut residual protein hydrolysate prepared under the optimal condition, identified on the basis of analyzing the key marker of cancer cells proliferation, and elucidated the possible mechanism underlying the activity on the breast cancer cell model, MCF-7.

## Methods

### Materials and chemicals

Walnuts were obtained from Hebei Jingpin Fruits Co., Ltd (Hebei, P.R. China). Proteases were purchased from Sigma-Aldrich Co. (St. Louis, MO, USA). Cell lines of MCF-7 (human breast cancer cell line), Caco-2 (human colon cancer cells), Hela (human cervical cancer cells) and IEC-6 (rat small intestinal crypt epithelial cells) were obtained from the Cell Culture Centre of the Institute of Basic Medical Sciences, Chinese Academy of Medical Sciences (Beijing, P.R. China).

### Preparation of different walnut residual protein hydrolysates

Walnuts were ground and defatted with petroleum ether. The defatted flour was dried in the drying oven overnight at 50 °C as walnuts oil extraction residue. Walnut residual protein (WRP) was extracted from walnuts oil extraction residue by NaOH solution (pH 9.0) at ratio 1:15 (w/v) for 1 h (45 °C). After pH adjustment to 4.5, the precipitate obtained by centrifugation at 4000 × g for 15 min and the supernatants were lyophilized. The protein hydrolysates were prepared with alkaline protease, papain, pepsin, trypsin and neutral protease, respectively. WRP was dissolved in distilled water at a concentration of 30 mg/ml and hydrolyzed for 3 h separately using alkaline protease at pH 9.0, 50 °C, papain at pH 7.0, 60 °C, pepsin at pH 2.5, 37 °C, trypsin at pH 8.0, 40 °C and neutral protease at pH 7.5, 55 °C. Samples were taken out after 3 h and heated in a boiling water bath at 95 °C for 10 min to inactive enzyme’activity. The samples were then centrifuged at 4000 × g for 15 min and the supernatants were lyophilized.

### Ultrafiltration

The protein hydrolysates were sequentially filtered through cellulose membranes (Ultrafiltration system Millipore, Bedford, Mass., USA) with molecular weight cutoffs (MWCO) of 10, 5, and 3 kDa (Millipore). Peptide fractions derived from walnut protein hydrolysates were: 10–5 kDa, 5–3 kDa and < 3 kDa fraction, respectively. All permeates were lyophilized and stored at −20 °C until further analysis.

### Cell culture

MCF-7 and Hela cells were grown in Dulbecco’s Modified Eagle’s Medium (DMEM) (Gibco, Rockville, MD, USA) containing 10 % heat-inactivated fetal calf serum (FCS) (Hyclone) and 0.5 % penicillin/streptomycin at 37 °C in a humidified atmosphere containing 5 % CO_2_. Caco-2 and IEC-6 cells were maintained in Dulbecco’s modified Eagle’s medium (DMEM, Gibco, Rockville, MD, USA)) supplemented with 10 % FCS (Hyclone), 0.1 mM nonessential amino acid, 0.5 % penicillin/streptomycin at 37 °C in a humidified atmosphere containing 5 % CO_2_.

### Cell Viability Assay

Cell viability was assessed by MTT assay [19]. Briefly, cells were seeded into 96-well microplates at a density of 3 × 10^4^ cells per well. After overnight incubation, cells were treated with various concentrations of samples and cultured for 48 h. After incubation, 20 μl of MTT (3-(4,5-dimethylthiazol-2-yl)-2,5-diphenyl-tetrazolium bromide, 5 mg/mL) reagent was added to each well and incubated for 4 h at 37 °C in the dark. The culture medium containing the MTT solution was replaced by 200 μL DMSO (dimethyl sulfoxide) and shaken in the dark for 15 min at room temperature for complete dissolution of the MTT formazan productions. The optical density of each well was measured by absorbance at 570 nm using a Benchmark Plus microplate reader (Bio-Rad, Hercules, USA). The cell viability and inhibitory rate was calculated as follows: Cell Viability (%) = *b/a* × 100; Inhibitory Rate (%) = [(*a* − *b*)*/a*] × 100, Where *a* is the optical density without sample, and *b* is the optical density in the presence of sample. The IC_50_ value is the concentration that inhibits 50 % of the cell viability.

### Gel filtration chromatography

The fraction with the highest antioxidant activity after ultrafiltration separation was re-dissolved in phosphate buffer(pH 7.0), then separated using a HiPrep 16/60 Sephacryl S-100HR column (16 × 600 mm, Amersham Pharmacia Biotech, Piscataway, NJ, USA) which was eluted with phosphate buffer at a flow of 1.0 ml/min, and monitored at 280 nm. The fractions with the desired peaks were pooled, desalted using a HiPrep™ 26/10 desalting column (Amersham Pharmacia Biotech, Piscataway, NJ, USA) and lyophilized for MTT assay.

### RP-HPLC

The fraction exhibiting the highest cytotoxic activity after gel filtration chromatography was further purified on a Zorbax SB-C18 (250 mm × 4.6 mm,5 um,Agilent,USA). The column was eluted by a linear gradient of acetonitrile (0-40 %) containing 0.1 % Trifluoroacetic acid (TFA) at a flow rate of 1.0 ml/min. The eluted peaks were detected at 214 nm and the fraction was then lyophilized.

### Molecular mass and amino acid sequence analysis

Accurate molecular mass and amino acid sequence of the active peptide was determined by Hybrid Quadrupole TOF-LC/MS/MS mass spectrometer (AB Sciex Instruments, CA, USA) coupled with ESI source. Generally, the peptide was infused into electrospray source following dissolve in acetonitrile/water (1:1, v/v), and molecular mass was determined by charged (M + H)^+^ state in the mass spectrum. Following molecular mass determination and sequence information was obtained by BioLynx analysis system.

### Simulated gastrointestinal digestion of bioactive peptides

The pH of the sample was adjusted to pH 2.0 by 1 N HCl, and the mixture was incubated with pepsin at 800 U/mL in a shaking bath for 2 h at 37 C with shaking at 100 rpm. The sample was then neutralized with 1 M NaOH to pH 7.0 before addition of pancreatin at 2 mg/mL and bile extract mixtureat 0.3 mg/mL for a further incubation of 2 h. Finally, the digestion was terminated by boiling for 10min [20].

### Cell cycle distribution analysis

MCF-7 cells were treated with or without test sample for 48 h. Cells were harvested fixed with cold ethanol/destillata (3:1, v/v) at 4 °C overnight. Before analysis, cells were washed with PBS and stained with a solution containing 100 μL propidium iodide, 100 μg/mL RNase at 37 °C for 30 min in the dark. The fluorescence of stained cells was analyzed by flow cytometry using a FACSCanto (Becton-Dickinson, CA, USA).

### Annexin V-FITC/PI double staining assay

Apoptosis can be detected by translocation of phosphatidyl serine to the cell surface using an annexin V-FITC antibody. MCF-7 cells were seeded in 6-well plates by density of 3 × 10^4^ cells/ml. Cells were treated with or without test sample for 48 h. The cells were counted after trypsinization and washed twice with cold PBS. The cell pellet was resuspended in 100 μl of binding buffer (10 mM HEPES, pH 7.4, 140 mM NaCl, and 2.5 mM CaCl_2_) at a density of 1 × 10^5^ cells per ml and incubated with 5 μl of FITC-conjugated Annexin-V and 10 μl of propidium iodide (PI) for 15 min at room temperature in the dark. Annexin V-FITC and PI fluorescence was monitored by flow cytometry (FACSAria, Becton–Dickinson, CA, USA). Cells that stained positive for Annexin V were classified as early apoptotic cells; cells positive for both PI and Annexin V staining were classified as late apoptotic cells; and PI and Annexin V negative cells were classified as live cells [19].

### TUNEL (TdT-mediated dUTP nick end labeling) assay

TUNEL technique has been extensively used for the detection and quantification of apoptosis. MCF-7 cells were cultured on coverslips and then fixed with stationary liquid (Anhydrous ethanol: chloroform: glacial acetic acid 6: 3:1), and digested in proteinase K for 20 min at 37 °C, before endogenous peroxidase was blocked in 3 % hydrogen peroxide. Terminal deoxynucleotidyl transferase (TdT) in reaction buffer (containing a fixed concentration of digoxigenin-labelled nucleotides) was applied to sections for 30 min at 37 °C, before the slides were placed in Stop/Wash buffer for 10 min. Following washes, a prediluted peroxidase conjugated antibody was applied for 30 min. Apoptotic cells were detected after incubation in the 3,3-diaminobenzidine (DAB) chromogen for approximately 10 min and slides were counterstained with hematoxylin [21, 22].

### Western blotting

MCF-7 cells were seeded in 6 cm dishes overnight and incubated with test sample for the 48 h. Cells were harvested by trypsinization and washed twice with cold PBS. Total cellular protein lysates were prepared by harvesting cells in protein extraction buffer (50 mM Tris–HCl, pH 7.4, 1 mM NaF, 150 mM NaCl, 1 mM EGTA, 1 mM phenylmethanesulfonyl fluoride; 1 % NP-40; and 10 mg/mL leupeptin) to the cell pellets on ice for 30 min, followed by centrifugation at 10,000 × g for 30 min at 4 °C. Equal amounts of protein (50 mg) were separated by 10 % sodium dodecyl sulfate–polyacrylamide gel electrophoresis (SDS-PAGE). After electrophoresis, proteins were electroblotted to nitrocellulose membranes, and subsequently incubated in blocking buffer (in PBS, 5 % skim milk, and 0.1 % Tween 20) at room temperature for 2 h. After blocking, the membranes were incubated with the antibodies, rabbit anti-LC3 I/II (1:800), and glyceraldehyde 3-phosphate dehydrogenase (GAPDH, 1: 800). Subsequently, membranes were washed five times in a wash buffer and incubated with appropriate donkey anti-rabbit horseradish peroxidase conjugated secondary antibody (1:8000), and the bands were disposed with the Gel-Pro Analyzer 4.0 (Media Cybernetics, USA) [19, 23].

### Immunofluorescence for LC3-II

MCF-7 cells were seeded on coverslips overnight and then incubated with test sample or rapamycin (RAPA) for the next 48 h. The cells fixed with 4 % (v/v) paraformaldehyde for 30 min and then covered with 10 % (v/v) goat serum for 60 min at room temperature followed by incubation with primary antibody of LC3-II in 1: 200 dilution at 4 °C overnight. Cells were then probed with Alexa Fluor 488-goat anti-rabbit IgG secondary antibody (1:200, Invitrogen Life Technologies, Carlsbad, CA, USA). Counterstaining of cell nuclei was performed by Vectashield H-1200 mounting medium with DAPI. Fluorescent staining was examined with a confocal fluorescence microscope (Nikon EZ-C1, Nikon, Tokyo, Japan). [24–26].

### Measurement of cytokine production

For cytokine immunoassays, Mouse spleen lymphocytes were cultured for 24 h at a density of 1 × 10^6^ cells/ml in 96-well plates. Supernatants were removed at the indicated time, and IL-2 production was quantified by sandwich immunoassays using the protocol supplied by ELISA kits (Beyotime Biotechnology, China).

### Neutral red uptake assay for macrophage phagocytosis

Macrophages were prepared from Kunming mice as described Chen et al. [27]. The phagocytic ability of macrophage was measured by neutral red uptake. After cells were cultured with test substances for 48 h, 100 μl neutral red solutions (dissolved in 10 mM PBS with the concentration of 0.075 %) was added and incubated for 1 h. The supernatant was discarded and the cells in 96-well plates were washed with PBS twice to remove the neutral red that was not phagocytized by RAW264.7 cells. Then cell lysate (ethanol and 0.01 % acetic acid at the ratio of 1:1, 100 μl/ well) was added to lyse cells. After cells were incubated in room temperature overnight, the optical density at 540 nm was measured by a micro-plate reader (Bio-Rad, Hercules, U.S.A.).

### Nitric oxide (NO) production by macrophages

Measurement of nitrite in medium was used as an indicator of NO production. The macrophage cells (5 × 10^5^ cells/ml) were cultured in 96-well plates with test substances. After 24 h, culture supernatants were collected and nitrite, the stable reaction product of NO with molecular oxygen, was measured using Griess reagent. Equal volumes of Griess reagent and sample were incubated together at room temperature for 10 min. Nitrite production was determined by comparing the absorbance at 540 nm with a standard curve generated by NaNO_2_ [20, 28].

### Statistical analysis

The data were expressed as means ± S.D. The significance of difference was evaluated with one-way ANOVA, followed by Student’s t- test to statistically identify differences between the control and treated groups. Significant differences were set at *p < 0.05, **p < 0.01, and ***p < 0.001.

## Results

### Cytotoxic activity of walnut protein hydrolysates on MCF-7 cells

**Fig. 1:**
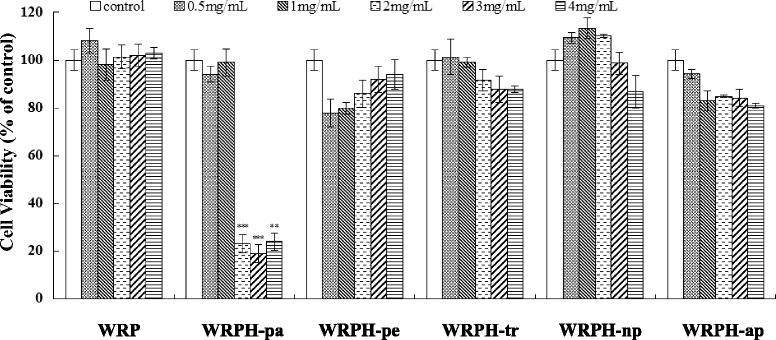
Effects of walnut residual protein and the enzyme protein hydrolysates on MCF-7 cell proliferation. After treated with test substances (0.5, 1, 2, 3, 4 mg/ml) for 48 h, cell viability was measured by MTT assay. WRP, walnut residual protein; WRPH-pa, walnut residual protein hydrolysate prepared with papain; WRPH-pe, walnut residual protein hydrolysate prepared with pepsin; WRPH-tr, walnut residual protein hydrolysate prepared with trypsin; WRPH-np, walnut residual protein hydrolysate prepared with neutral protease; WRPH-ap, walnut residual protein hydrolysate prepared with alkaline protease. Results were expressed as means ± S.D. of four separate experiments. Statistical significance test for comparison with control (untreated) group was done by Student’s *t*-test. *p < 0.05; **p < 0.01; ***p < 0.001

To investigate whether anticancer bioactive peptide is contained in walnut, WRP was hydrolyzed separately by papain, pepsin, trypsin, neutral protease and alkaline protease as described above, and termed as WRPH-pa, WRPH-pe, WRPH-tr, WRPH-np and WRPH-ap respectively. The effect of walnut residual protein and the protein hydrolysates on proliferation of MCF-7 cells was investigated by MTT cell proliferation assay. As shown in Fig. 1, the results indicated that the protein hydrolysates inhibited cells growth, while WRP was inert. Among all hydrolysates, WRPH-pa showed significant cytotoxic activity in a dose-dependent manner, and the IC50 value was 1.62 mg/ml after 48 h treatment, while the other four different enzymatic protein hydrolysates (WRPH-pe, WRPH-tr, WRPH-np and WRPH-ap) showed no obvious inhibitory effect (Fig. 1). This result suggested that bioactive peptide(s) with cytotoxic activity on the cancer cells was (were) contained in walnut residual protein, and was (were) released during papain hydrolysis.

**Fig. 2:**
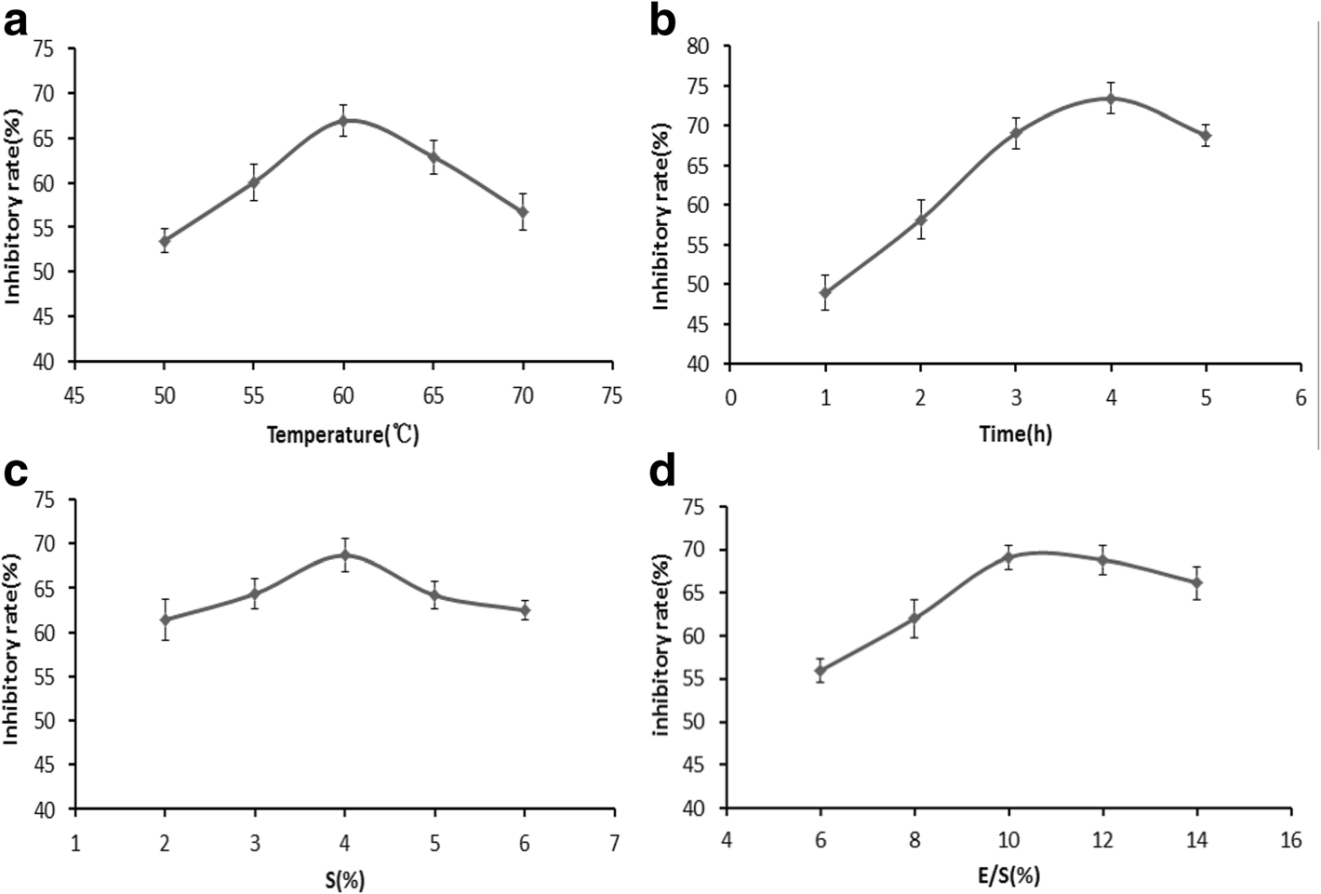
Effect of WRPH-pa in different hydrolytic conditions on MCF-7 cell proliferation. **a** WRPH-pa was hydrolyzed at temperature of 50, 55, 60, 65 and 70 °C, respectively with the conditions of time 3 h, substrate concentration 4 % and an enzyme-substrate ratio 10 %. **b** WRPH-pa was hydrolyzed at time of 1, 2, 3, 4 and 5 h, respectively with the conditions of temperature 60 °C, substrate concentration 4 % and an enzyme-substrate ratio 10 %. **c** WRPH-pa was hydrolyzed at substrate concentrations of 2, 3, 4, 5 and 6 %, respectively with the conditions of temperature 60 °C, time 3 h and an enzyme-substrate ratio 10 %. **d** WRPH-pa was hydrolyzed at enzyme-substrate ratio of 8, 9,10,11 and 12 %, respectively with the conditions of temperature 60 °C, time 3 h and substrate concentration 4 %. WRPH-pa, walnut residual protein hydrolysate prepared with papain

Various parameters play a great role in the optimization of the experimental conditions for hydrolyzing protein. Temperature (T), time (t), enzyme-substrate ratio 330 (E/S) and substrate concentration (S) are generally considered to be the most important factors that affect protein hydrolysis. The investigated levels of each factor were selected depending on the MTT cell proliferation assay result of the single-factor (Fig. 2). Independent variables with three variation levels, X1 (temperature: 55, 60, 65 h), *X*2 (time (t): 3, 4, and 5 h), X3 (substrate concentration: 3, 4, and 5 %) and X4 (enzyme-substrate ratio: 8, 10, and 12 %) are presented in Table 1. Orthogonal experimental design was implemented to illustrate the effects of different factors involved in the enzymatic hydrolysis. The results of the orthogonal test are showed in Table 1. The results of the orthogonal test are showed in Table 1. The factors affecting the cytotoxic activity of WRPH-pa are listed in descending order according to the range values (R): D > A > C > B. The enzyme-substrate ratio used during the enzymolysis was found to be the most important factor. The WRPH-pa with the higher cytotoxic activity was prepared in the condition of D2A2C3B1. These results indicated that walnut residual protein hydrolysates with the higher cytotoxic activity on MCF-7 cells could be prepared by papain in the condition as follow: 5 % substrate concentration and an enzyme-substrate ratio of 10 % at temperature 60 C for 3 h.

**Table 1:** Results and analysis of the orthogonal test

No.	A	B	C	D	IR (%)^a^
	Temperature (°C)	Time (h)	S (%)	E/S (%)	
1	55	3	3	8	66.13 ± 0.78
2	55	4	4	10	69.57 ± 1.13
3	55	5	5	12	63.73 ± 1.83
4	60	3	4	12	66.41 ± 0.85
5	60	4	5	8	70.55 ± 0.69
6	60	5	3	10	68.21 ± 0.50
7	65	3	5	10	68.34 ± 2.15
8	65	4	3	8	60.53 ± 1.90
9	65	5	4	12	64.81 ± 1.79
K1	66.48 ± 2.94	66.96 ± 1.20	64.96 ± 3.97	65.74 ± 5.02	
K2	68.39 ± 2.08	66.89 ± 5.52	66.93 ± 2.42	68.71 ± 0.75	
K3	64.56 ± 3.91	66.58 ± 2.34	67.54 ± 3.48	65.98 ± 1.35	
Best level	60 °C	3 h	5 %	10 %	
R	3.67	0.60	1.82	3.88	
R order	D > A > C > B				

^a^MCF-7 inhibitory rate

### Identification of bioactive peptides with cytotoxic activity on MCF-7 cells

**Fig. 3:**
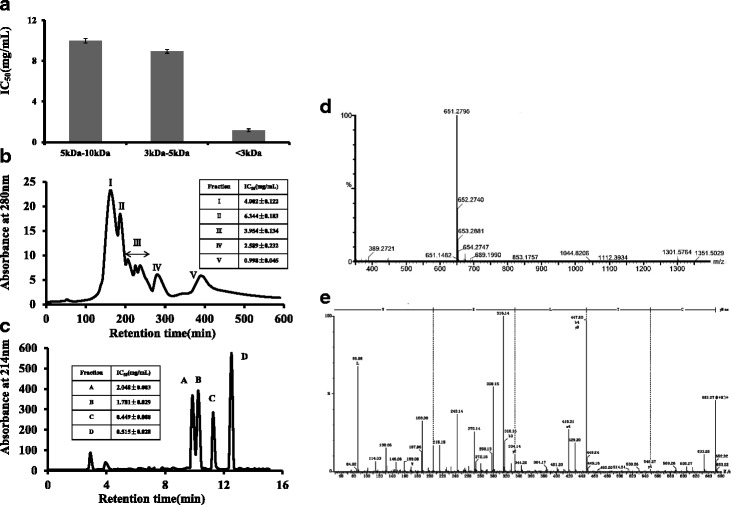
Isolation of bioactive peptides with cytotoxic activity on MCF-7 cells. **a** IC_50_ value of WRPH-pa with different molecular weights on MCF-7 cell proliferation. **b** Gel filtration chromatography of WRPH-pa with <3 kDa molecular weights on a HiPrep 16/60 Sephacryl S-100HR column. Insets show the IC_50_ value of fraction I to V on MCF-7 cell proliferation. **c** RP-HPLC chromatography of fraction V from gel chromatography. Insets show the IC_50_ value of fraction A to D on MCF-7 cell proliferation. **d**, **e** Identification of fraction C by TOF-LC/MS/MS coupled with ESI source. **d** Amino acid sequence analysis of the bioactive peptide. **e** Molecular mass analysis of the bioactive peptide. Results were expressed as means ± S.D. of four separate experiments

The peptide length and molecular weight distribution of hydrolysates were considered to be closely related to their biological activities [29, 30]. In most cases, low or relatively low molecular weight exhibited bioactivities [2, 11, 31, 32]. Peptides longer than 10 kD were found to have less or no cytotoxic activity on cancer cell growth [12]. Ultrafiltration membrane with MWCO 3 kDa, MWCO 5 kDa, MWCO 10 kDa was used to separate the WRPH-pa into three fractions, WRPH-pa1 (10–5 kDa), WRPH-pa2 (5–3 kDa) and WRPH-pa3 (<3 kDa). WRPH-pa3 with the lowest molecular weight showed the highest cytotoxic activity among the three fractions (Fig. 3a). Compared with the higher molecular weight peptides, the lower molecular weight peptides have greater molecular mobility and diffusivity, which seems to promote interactions with cancer cell components and improve anticancer activity [33]. WRPH-pa3 exhibited the higher cytotoxic activity was selected for further study. WRPH-pa3 was then separated into five fractions(I-V) by gel filtration (Fig. 3b). FractionVexhibited the higher cytotoxic activity(Fig. 3b Inset). The active fraction was further fractionated on a RPLC C18 column (Fig. 3c). Four fractions (A-D) were collected separately. While all of the fractions showed the cytotoxic activity, fraction C and D had the higher cytotoxic activity (Fig. 3c Inset). That is, the higher the hydrophobicity of peptides, the stronger the cytotoxicity against MCF-7 cells. Fraction C had the highest cytotoxic activity among the four fractions, the IC50 was 0.449 mg/ml (Fig. 3c Inset). Fraction C was then rechromatographed on RPLC C18 column. A single pure peptide was eventually obtained. The amino acid sequence of the peptide was identifies as CTLEW (Cys-Thr-Leu-Glu-Trp), and the peptide possesses a m/z value of 651.2795 (MH+, protonated mono isotopic mass) by TOF-LC/MS/MS and BioLynx analysis system. (Fig. 3d and e) The active peptide (CTLEW) managed to retain about 82.1 % of its original cytotoxic activity even after successive digestion with pepsin, bile extract and pancreatin (data not shown). Following simulated gastric and intestinal digestion, no significant difference was found in cytotoxic activity, which would indicate that the peptide CTLEW may survive in the process of gastrointestinal transit. These results showed that the bioactive peptide with cytotoxic activity on MCF-7 cells in walnut protein hydrolysates was a pentapeptide CTLEW with molecular weight of 651.2795 Da.

### CTLEW Induced apoptotic and autophagic cell death

**Fig. 4:**
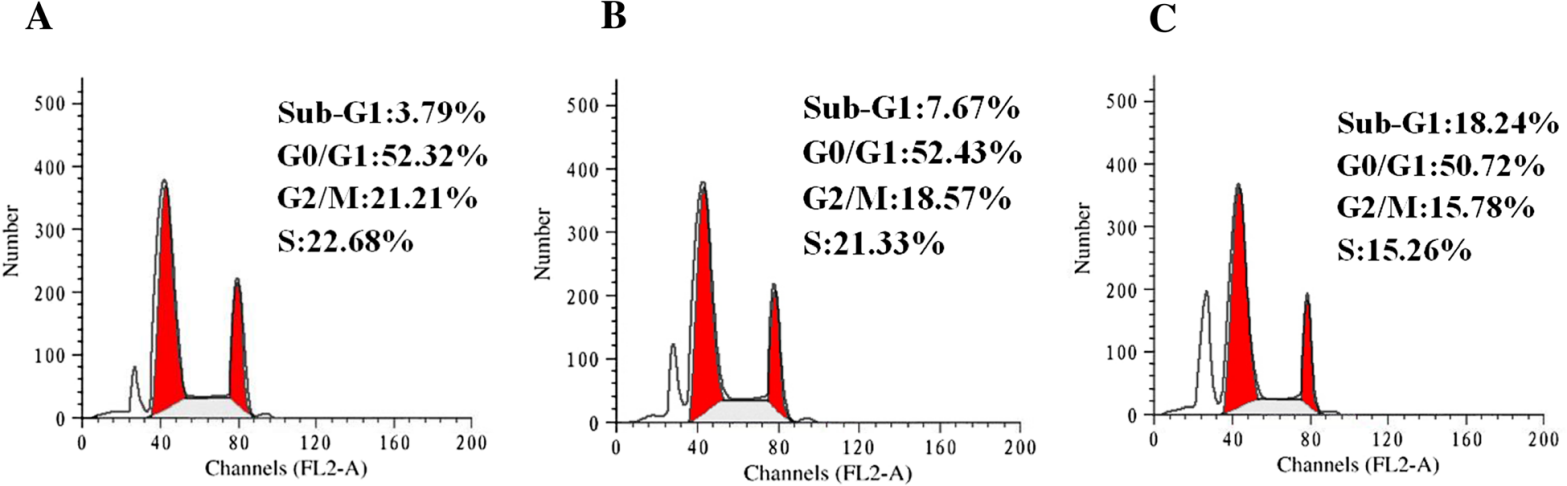
Effect of CTLEW on cell cycle distribution in MCF-7 cells. After treated with CTLEW (0, 0.5, 1.0 mg/ml) for 48 h, cells were stained by propidium iodide. The fluorescence of stained cells was analyzed by flow cytometry. **a** control (0 mg/ml of CTLEW); (**b**) 0.5 mg/ml of CTLEW; (**c**) 1.0 mg/ml of CTLEW. Data analysis was performed with ModFit LTTM cell cycle analysis software

Since CTLEW significantly inhibited the proliferation of MCF-7 cells, we then tried to investigate the possible mechanism underlying cytotoxic activity of CTLEW on growth of MCF-7 cells. Cell cycle distribution was analyzed by flow cytometry and apoptosis was assessed by Annexin V FITC/ PI double-staining and TUNEL assay. MCF-7 cells were treated with the indicated concentration of CTLEW for 48 h. As shown in Fig. 4, CTLEW significantly increased the number of cells in sub-G1 phase to 7.67 or 18.24 % compared with control (3.79 %) at the concentration of 0.5 or 1.0 mg/ml. These results suggested that CTLEW might be induced apoptosis of MCF-7 cancer cells in a dose-dependent manner.

**Fig. 5:**
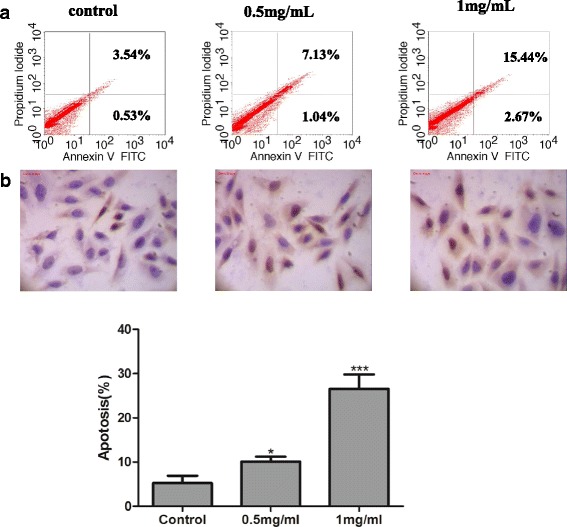
Induction of apoptosis cell death by CTLEW in MCF-7 cells. **a** Annexin V-FITC/PI doubles staning assay. Cells were treated with (0.5 or 1.0 mg/ml) or without (control) CTLEW for 48 h, and were stained by annexinV-FITC/PI, respectively. Phosphotidylserine externalization in MCF-7 cells was determined by flow cytometry of annexinV-FITC/PI stained cells. **b** TUNEL apoptosis assay. Cells were treated with (0.5 or 1.0 mg/ml) or without (control) CTLEW for 48 h, cell tissue slice was labelled by digoxigenin and observed by immunocytochemical staining, the labelled yellow brown nucleus represents the apoptosis cells. Data were presented as mean ± S.D. of three separate experiments

To determine apoptosis mediated the cytotoxicity of CTLEW, the loss of phosphatidylserine asymmetry, a hallmark of apoptosis, was quantified by cytometry of propidium iodide/Annexin V double-stained cells [26]. As shown in Fig. 5a, Annexin V FITC/ PI double-staining assay indicated that CTLEW induced phosphatidylserine externalization without increasing the proportion of propidium iodide cells, implicating that CTLEW induced apoptosis but not necrotic cell death. The total apoptosis rates were 8.17 and 18.11 % (control, 4.07 %) in the cells treated with CTLEW at the concentration of 0.5 and 1.0 mg/ml. Apoptosis was further confirmed by TUNEL assay. Treating MCF-7 cells with various concentrations of CTLEW (0.5 or 1.0 mg/ml) for 48 h, cell tissue slices were observed by immunocytochemical staining, where the labelled yellow brown nucleus represented the apoptotic cells. As shown in Fig. 5b, apoptosis rate were 10 and 25 % (control, 5.0 %), respectively, in the cells treated with CTLEW at the two concentrations. These findings suggested that CTLEW induced apoptosis in a dose-dependent manner.

**Fig. 6:**
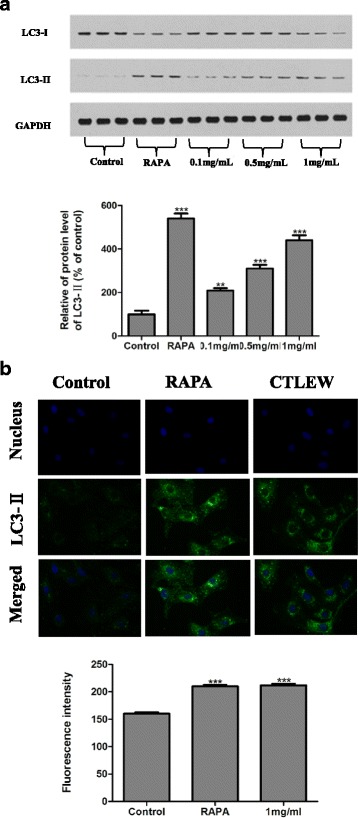
Induction of autophagic cell death by CTLEW in MCF-7 cells. **a** Western blotting of LC3I/II. Cells were treated with (0.1, 0.5, and 1.0 mg/ml) or without (control) CTLEW for 48 h, and expression of LC3I/II in MCF-7 cells was detected by Western blotting. GAPDH was used as internal control and RAPA was used as a positive control. **b** Immunofluorescence for LC3-II. Cells were treated with (1.0 mg/ml) or without (control) CTLEW for 48 h, and formation of autophagic vacuoles was detected by immunofluorescent staining for LC3-II. RAPA was used as a positive control. Blue, DAPI-stained cell nuclei; Green, LC3-II-stained cytoplasm (100x magnification). Fluorescence intensity represents the expression of LC3-II. Data were presented as mean ± S.D. of three separate experiments. *p < 0.05; **p < 0.01; ***p < 0.001, significantly different from the respective control group

To evaluate autophagic cell death induced by CTLEW, LC3I/II was determined by western blot analysis. As shown in Fig. 6a, CTLEW significantly increased the level of phosphatidylethanolamine-conjugated LC3-II, whereas unconjugated LC3-I levels were slightly decreased. The result indicated the activation of autophagic cell death in CTLEW treated cells. Autophagy was further confirmed by immunofluorescence for LC3-II. Immunofluorescent staining was observed with a confocal fluorescence microscope. The activation of LC3II was revealed in the cytoplasm in CTLEW -treated MCF-7 cells (Fig. 6b). Furthermore, LC3-II protein expression was increased remarkably upon treatment with 1.0 mg/ml of CTLEW, which was comparable to that of RAPA, again suggesting the CTLEW -induced cells autophagy (Fig. 6b). Taken together, these results showed that CTLEW was able to induce apoptosis and autophagy in MCF-7 cells.

### Effect on different cell types and immunomodulatory activity of CTLEW

To investigate effect of CTLEW on other cancer cells and non-cancer cells, cell viability was measured by MTT assay on Caco-2, Hela and IEC-6 cell lines respectively, after treated with the active peptide for 48 h. As shown in Table 2, the results indicated that CTLEW also significantly inhibited proliferation of Caco-2 (IC50 value 0.65 ± 0.42 mg/ml) and Hela (IC50 value 0.60 ± 0.17 mg/ml). Interestingly, the peptide showed almost no inhibition on the growth of IEC-6 (IC50 value >4 mg/ml, the highest concentration treated cells was 4 mg/ml). These results showed that CTLEW could kill cancer cells selectively, and was less toxicity to normal cells of IEC-6. CTLEW from walnut by-products could be as an anticancer peptide.

**Table 2:** Effect of CTLEW on other cancer and normal cell lines

Cell lines	Hela	Caco-2	IEC-6	spleen lymphocytes
IC50/(mg/mL)	*0.60 ± 0.17	*0.65 ± 0.42	*>4	*>4

*After treated with CTLEW(0.5,1,2,3,4 mg/ml) for 48 h, cell viability was measured by MTT assay. The IC_50_ value is the concentration that inhibits 50 % of the cell viability, compared with control(untreated with CTLEW)

**Fig. 7:**
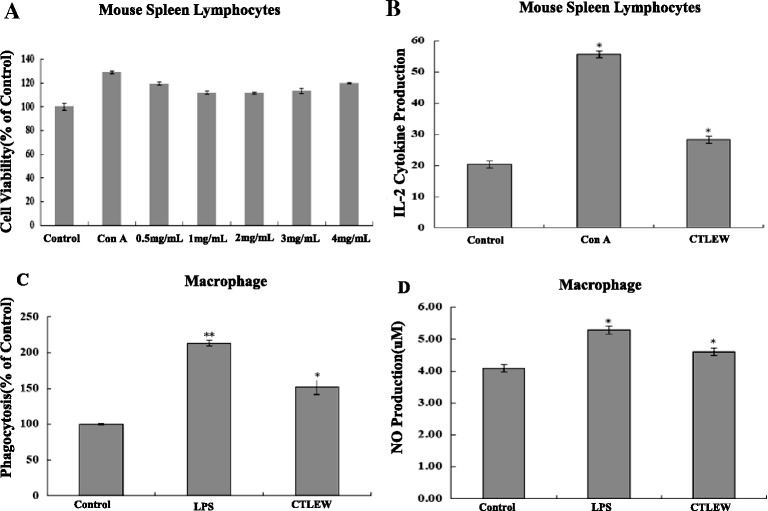
Immunomodulatory activity of CTLEW. **a** Effects of CTLEW on mouse spleen lymphocytes proliferation. Cells were pretreated with (0.5, 1, 2, 3, 4 mg/ml) or without (control) CTLEW for 48 h. Con A (10 μg/ml) was used as a positive control. Cell viability was measured by MTT assay. **b** Effects of CTLEW on IL-2 cytokine production in mouse spleen lymphocytes. Cells were cultured for 24 h in the media with (0.5 mg/ml) or without (control) CTLEW. ConA (10 μg/ml) was used as a positive control. The amounts of IL-2 released into the culture media were measured by immunoassays. **c** Effect of CTLEW on phagocytosis of macrophages by neutral red uptake assay. After treated with (0.5 mg/ml) or without (control) CTLEW for 48 h, macrophage phagocytosis was determined by Neutral red uptake assay. LPS (1 μg/ml) was used as a positive control. (D), Effect of CTLEW on the NO production of macrophages. Cells were pretreated with (0.5 mg/ml) or without (control) CTLEW for 24 h. LPS (1 μg/ml) was used as a positive control. The supernatant nitrite levels were determined using Griess reagent. Results were expressed as means ± S.D. of four separate experiments. Statistical significance test for comparison with untreated group was done by Student’s *t*-test. *p < 0.05; **p < 0.01, ***p < 0.001

To assess the immunomodulatory activity of CTLEW, spleen lymphocyte and macrophage functions were examined. MTT assay of spleen lymphocyte cultured in the medium with or without CTLEW showed that it enhanced the cells proliferation at all concentrations, showed immunostimulatory activity in a dose-independent manner (Fig. 7a). The effect of CTLEW on IL-2 production by spleen lymphocyte was measured by ELISA. Compared with control, IL-2 production of spleen lymphocyte treated by the peptide significantly increased (Fig. 7b). In line with the spleen lymphocyte proliferation results, CTLEW exhibited immunostimulatory effect on spleen lymphocyte. Phagocytosis and NO production were the main channels for macrophages to play the immunological effect. To determine the effect of the peptide on the phagocytic activity of macrophage, the neutral red uptake assay was performed. Macrophages incubated in culture medium with or without the active peptide for 48 h were used to test the phagocytosis. As shown in Fig. 7c, CTLEW showed a significant activity of promoting macrophages to devour neutral red. NO production of the cells was determined by Griess assay. After cells were incubated with CTLEW for 24 h, NO level of the experimental groups was significantly elevated compared to the control group (Fig. 7d). These results indicated that the anticancer peptide also possessed potential immunomodulatory effect.

## Discussions

Generally, bioactive peptides are inactive within the sequences of their parent proteins, and need to be released through enzymatic hydrolysis [11, 16]. A wide variety of smaller peptides are generated via hydrolysis, depending on enzyme specificity and hydrolytic time [2]. The difference of enzymes selected usually leads the hydrolysates to have different qualities and bioactivities due to different amino acid sequences and peptide lengths [18]. In this study, walnut residual protein did not show any impair towards MCF-7 cancer cell growth, but its hydrolsates formed by commercial proteases including papain, pepsin, trypsin, neutral protease and alkaline protease exhibited inhibitory activity against the tumor cell lines. Compared to hydrolyzed walnut residual proteins from other four enzymes, papain-derived hydrolysates exhibited the strongest cytotoxic activity. It is clear that the anticancer peptide contained in walnut residual protein can be released and well-prepared by papain hydrolysis. The optimized conditions for the manufacture of the walnut residual protein hydrolysates which have higher cytotoxic activity against MCF-7 cells by papain are 5 % substrate concentration and an enzyme-substrate ratio of 10 % at temperature 60 °C for 3 h incubation time.

Papain as an active endolytic cysteine protease does not exhibit a broad specificity for peptide bonds, but exhibits a preference in an amino acid bearing a large hydrophobic side chain [34, 35]. Thus, the amino acid sequence of the active peptide isolated from the papain-forming hydrolysates is obtained as CTLEW throughout sequential ultra-filtration, gel filtration chromatography and RP-HPLC. C (Cys) is a hydrophilic amino acid, and W (Trp) is a hydrophobic amino acid. Cys and Trp arranged in two sides of the peptide of CTLEW form clear hydrophilic and hydrophobic surfaces. Obviously, CTLEW is a peptide with an amphiphilic structure. Our results of MTT assay on Caco-2, Hela and IEC-6 cells indicated that CTLEW could kill cancer cells selectively, and was less toxicity to normal cells of IEC-6. The high amphipathic tend to be the heart of anticancer activity [17]. The amphiphilic structure is conducive for the membrane binding of anticancer peptides [36]. Amphiphilicity levels and hydrophobic arc size are of major importance to the ability of these peptides to invade cancer cell membranes [37]. W (Trp) arranged in the sides of the active peptide is the amino acid with higher hydrophobic arc size. It is reported that the spatial position and arrangement of tryptophanes (W) affect membrane-active peptide adsorption and activity [38, 39] and tryptophanes have been observed to modulate hydrophobic mismatches to maintain peptide stability and activity in lipid bilayer membranes [40]. In addition, anticancer peptides are generally stabilized by the disulfide bonds. Defensins, lactoferricin, and tachyplesin are constrained by disulfide bonds [17]. C (Cys) residues create a disulfide bond linking the highly positively charged NH2-terminal region and the COOH-terminal region of the peptide [41]. Taken together,CTLEW peptide isolated from walnut residual protein by papain hydrolysis is an amphiphilic structure,might be conducive to invade cancer cell membranes, and might be possessed higher hydrophobic arc size and stabilized by the disulfide bonds due to C and W arranged in two sides of the active peptide. CTLEW from walnut residual protein should be regarded as a novel anticancer peptide, science it has not been reported so far.

To investigate the possible mechanism underlying cytotoxic activity of CTLEW on growth of MCF-7 cells, Cell cycle distribution, Annexin V FITC/ PI double-staining and TUNEL assay were performed. CTLEW significantly increased the number of cells in sub-G1 phase, induced phosphatidylserine externalization without increasing the proportion of propidium iodide cells, implicating that it induced apoptosis. Apoptosis was further confirmed by TUNEL assay observed the apoptotic cells. Besides apoptosis, autophagy has been described as an alternative self-destructive cellular process, which is linked to cancer cell death [42]. Apoptotic and autophagic cell death are not mutually exclusive pathways, they could induce cell death simultaneously and cooperatively [42, 43]. Apoptotic process could be controlled by autophagy to be more probable, and vice versa [44]. Autophagy, an evolutionarily conserved process, regulates cell death in both physiological and pathophysiological conditions, in which cytoplasmic components including entire organelles are targeted for lysosomal degradation [45, 46]. The microtubule associated protein 1 light chain 3 (LC3) plays a critical role in autophagy [46, 47]. It normally resides in the cytoplasm as LC3-I, which associates with autophagosomal membranes during macroautophagy after modification to LC3-II by attachment of phosphatidylethanolamine [48, 49]. The conversion of the soluble form of LC3-I to the autophagic vesicle-associated form LC3-II is considered a specific marker of autophagosome promotion [50]. Therefore LC3-II is one of the principle cell death markers that correlate with the extent of autophagy [24, 26]. LC3I/II was determined by western blot analysis, CTLEW significantly increased the level of phosphatidylethanolamine-conjugated LC3-II, whereas unconjugated LC3-I levels were slightly decreased, indicating the active peptide induced autophagic cell death. Autophagy was further confirmed by immunofluorescence for LC3-II. CTLEW exhibited cytotoxic activity on cancer cells by inducing apoptosis and autophagy.

Autophagy is a caspase independent cell death pathway [51], and co-activation of autophagy and caspase independent apoptosis has been documented in various biological contexts [52–54]. Anticancer Peptide FK-16 induced caspase-independent apoptosis and autophagy through the common p53-Bcl-2/Bax cascade in colon cancer cells [26]. In the present study, the apoptosis induced by CTLEW might be a caspase independent cell death pathway induced by the decrease in mitochondrial membrane potential and release of cytochrome c from mitochondria to cytosol [55]. In another hand, several studies established nitric oxide NO as an effector of apoptosis [53, 54]. Our results of Griess assay showed that production of intracellular NO in MCF-7 cells treated with CTLEW was increased in a dose-dependent manner (data not shown). NO, as a free-radical gas, is synthesized by inducible nitric oxide synthase (iNOS) [56]. Secretion of NO was through stimulating intracellular signaling molecules such as MAPKs [57]. Earlier reports suggest that JNK signaling is of primary importance in controlling iNOS expression. Avik et al. report suggested that iNOS- mediated production of NO was identified as an effector molecule causing apoptosis of cancer cells, expression of iNOS was induced by activation of JNK and ERK subsequently [55]. CTLEW inducing apoptosis and autophagy might be in caspase independent manner, and nitric oxide NO might be as an effector of apoptosis.

Recent studies have revealed immunomodulatory properties of anticancer peptide from soybean,frog skin and other food [9, 58]. The results of immunocyte functions assay showed that the anticancer peptide enhanced proliferation and IL-2 secretion of spleen lymphocytes, promoted phagocytosis and NO production of macrophages, suggesting the anticancer peptide revealed immunostimulatory effect. Previous studies have shown that antioxidants, such as peptide isolated in soybean, lunasin [9] or phenolics from emblica fruit [59] possessed anticancer and immunomodulatory activity simultaneously, because cancer cells have long been known to have higher oxidative stress than their normal counterparts [55]. However, manipulation of intracellular oxidative stress either by antioxidants [60] or by pro-oxidants [61, 62] has also been exploited for preferential killing of cancer cells. To investigate the antioxidant activity of the active peptide, DPPH, hydroxyl radical scavenging and reducing power assays of CTLEW were performed. However, the results showed that the active peptide did not reveal antioxidant activity (data not shown), indicating that the anticancer and immunomodulatory activity were not associated with the antioxidant effect.

## Conclusions

This work uses papain firstly to degrade WRP to produce anticancer peptide. The anticancer peptide is a novel bio-peptide with the amino acid sequence CTLEW, and its molecular weight is 651.2795 Da with an amphiphilic structure. CTLEW shows a selective inhibition towards cancer cells by inducing apoptosis and autophagy, and is less toxicity to non-cancerous cells. Moreover, the bio-peptide indicates potential immunomodulatory effect. The optimized conditions for the release of CTLEW from WRP are 5 % substrate concentration and an enzyme-substrate ratio of 10 % at temperature 60 °C for 3 h incubation time. These results suggested that WRP from oil process can be applied to produce an anticancer peptide via adequate hydrolysis, the hydrolysates and/or its isolated peptides may be effectively used as functional food ingredients and/or pharmaceuticals.

## Abbreviations

CTLEW: Cys-Thr-Leu-Glu-Trp
INOS: Inducible nitric oxide synthase
LC3: Light chain 3
MTT: (3-(4, 5-dimethylthiazol-2-yl)-2, 5-diphenyl-tetrazolium bromide
NO: Nitric oxide
TUNEL: TdT-mediated dUTP nick end labeling
WRP: Walnut residual protein
WRPH-ap: Walnut residual protein hydrolysate by alkaline protease
WRPH-np: Walnut residual protein hydrolysate by neutral protease
WRPH-pa: Walnut residual protein hydrolysate by papain
WRPH-pe: Walnut residual protein hydrolysate by pepsin
WRPH- tr: Walnut residual protein hydrolysate by trypsin
WRPH-pa1: Walnut residual protein hydrolysate by papain with MW below 3 kDa
WRPH-pa2: Walnut residual protein hydrolysate by papain with MW 3–5 kDa
WRPH-pa3: Walnut residual protein hydrolysate by papain with MW 5–10 kDa
